# Japan renews primary health care to promote healthy ageing

**DOI:** 10.2471/BLT.18.030718

**Published:** 2018-07-01

## Abstract

Yoshitake Yokokura has been driving efforts to renew primary health care in Japan to meet the needs of an ageing population. He talks to Fiona Fleck.

Q: How did you become interested in medicine?

A: My father was a doctor in the village of Takata in the Fukuoka Prefecture. He would accept any patient who needed medical care. He did his best to educate people about infectious diseases and to be aware of the health status of members of the community. So health care was an important part of my life while I was growing up. At the age of 17, I had appendicitis. My uncle who was a surgeon did the operation. After that, the pain completely disappeared and I realized the power of health care and medicine. I was also inspired by my father and, like him, felt a strong desire to be of service to people.

Q: What were the challenges when you started working as a physician?

A: In the 1950s and 1960s, infectious diseases were highly prevalent in Japan, particularly tuberculosis, which was called “the national disease.” The Tuberculosis Prevention Act was passed in 1951 and anti-tuberculosis drugs became available. These developments contributed to a sharp reduction in tuberculosis deaths around the time I became a physician in 1969 and infectious diseases were at a turning point. Noncommunicable diseases (NCDs), such as heart disease, cancer and high blood pressure, were becoming more prevalent and physicians started to provide health check-ups to prevent and detect NCDs. Japan’s economy was growing rapidly and health problems related to industrial contamination and other forms of pollution emerged, for example, Minamata disease caused by mercury contamination and asthma associated with air pollution. A new public health movement sprung up in Japan that sought to do more research and prevent these pollution-related diseases. In addition, there was a large increase in traffic accidents owing to the progress of motorization, and we have a long history battling natural disasters, so emergency care has become very important in Japan and that’s also why I decided to become a surgeon.

Q: How has the work for physicians changed in Japan?

A: Today, the average life expectancy in Japan is in the 80s and, because we have a large elderly population, NCDs are highly prevalent. About one out of two Japanese people will develop cancer at some point in their lives, and since many cancers are treatable, one major challenge is to accommodate the many people on cancer treatment, so that they can continue to work and take their place in society. Due to ageing, we also face the challenge of dementia. Since 2015, the health sector has been implementing the government’s plan to address the challenge of dementia. The plan envisages a society in which dementia patients are respected and can continue to live at home and in the community for as long as possible. This should be achieved through the provision of timely and appropriate medical and nursing care, as well as support for the caregivers themselves,

“The plan envisages a society in which dementia patients are respected and can continue to live at home and in the community for as long as possible.”

Q: How are physicians adapting to an ageing society?

A: In Japan, most physicians set up a private practice after completing specialty training. However, our physicians are often concentrated in urban areas, while rural areas may be poorly served. Moreover, physicians need to address the demands of a rapidly ageing population. For these reasons, in 2012, the Japan Medical Association (JMA) started encouraging people to register with *kakaritsuke* physicians.

*Q: What is a *kakaritsuke *physician?*

A:* Kakaritsuke* physician does not refer to a qualification or position such as “general practitioner,” but a role or function played by a physician who may be a family doctor, a specialist or a hospital physician and who practices in his or her community. This is a physician who has up-to-date general medical knowledge, whom people can consult on any health issue and who can refer a patient to another specialist when needed. These physicians advise patients on how to stay healthy for as long as possible thus contributing to a society in which elderly people remain active. There are five key components to their work. First, they take a patient-centred approach to health care, attending to the individual as a whole. Second, they take a life-course approach, attending to patients at diverse time-periods, from the early years to old age. Third, they provide health care by teaming up with other professionals, for example, social workers to address the needs of the elderly. Fifth, they need to be able to fill in any health-care gaps. For example, if hospices are not available, then they need to be able to provide palliative care in patients’ homes.

*Q: What is the incentive for specialists to become *kakaritsuke* physicians?*

A: There are no specific financial incentives: *kakaritsuke* physicians are paid according to the standard medical fee schedule. However, physicians, including specialists, in Japan realize that patients have a wide range of needs that they can best meet as *kakaritsuke* physicians. Many elderly people have multiple diseases and health conditions, and visiting different specialists in many clinics and hospitals is a burden for some and not an option for others. These elderly patients are better off seeing or being visited by a single physician who knows them well. So there is a high demand for this type of physician. That is why the JMA started a training programme in 2016 to increase the capacity of *kakaritsuke* physicians’ to address the health needs of our aging society. Currently every year about 10 000 physicians including clinical specialists and former hospital physicians complete this training.

“Physicians, including specialists, in Japan realize that patients have a wide range of needs that they can best meet as kakaritsuke physicians.”

*Q: How do the* kakaritsuke *physicians meet the needs of Japan’s ageing population?*

A: The JMA training programme covers a wide range of age-related health problems from end-of-life care and dementia to polypharmacy, rehabilitation and nutrition. In the past, social care and medical treatment in Japan were separate, but many elderly people need both, so we are bringing these services together. That’s another reason why the *kakaritsuke* physicians have become so important, because they assess elderly patients for social care and medical needs.

Q: What is the JMA doing to protect physicians from over-work and burnout?

A: The Japanese government has undertaken workplace reform to address issues of employees spending longer than necessary at their workplace, which may cause productivity losses, poor health and exhaustion in the workplace including the health sector. Recently, the JMA conducted a survey of 10 000 physicians in response to such concerns and proposed “*Seven fundamentals for physicians to work energetically*,” for example “let’s get enough hours of sleep,” “let’s take at least one day off a week” and “let’s not try too hard” and “*Seven fundamentals for hospitals to protect the health of their physicians*,” such as providing a supportive and healthy working environment.

Q: Japan’s model of universal health coverage (UHC) has been praised by the World Bank, but criticized by analysts as financially unsustainable. What is being done to address this?

A: UHC was rolled out in Japan step by step. The Health Insurance Act was enacted in 1922, requiring companies to contribute to their employees’ health insurance. This social health insurance for employees was later extended to everyone else, with coverage provided by regional health insurance funds. The public health insurance programme is applied uniformly nationwide so that each individual has equal access to health-care services. To address concerns about financial sustainability, the JMA is promoting preventive medicine, encouraging approaches that preserve people’s health for as long as possible and the integration of health screening programmes.

Q: Given Japan’s ageing population and growing health care needs, what is the government doing to ensure financial sustainability of the health system?

A: In Japan, about 15 years ago, there was a big health-care budget cut – of 3.6% – and “health system disintegration” became a common expression. The health system survived, but many health-care workers took salary cuts, had to work longer hours and became very tired. With a smaller budget, fewer people could enter the health professions. Over the past six years, the budget for health services and health insurance has not increased, but workforce costs have increased by 1.6%, because of the increased demand for services. Moreover, we have not yet introduced cost-effectiveness to the extent of having an agency that assesses which health products and interventions offer the best value for money. Currently, the Japanese government is proposing increasing patient contributions to address the financial sustainability issue. If this proposal is implemented, the financial burden on patients, particularly the elderly, will increase significantly. The JMA opposes this proposal and, through negotiations with the government, we are working hard to protect the health of all patients without increasing the burden on them.

Q: As president of the World Medical Association (WMA) what are you trying to achieve?

A: Japan has one of the longest average life expectancies and one of the longest healthy life expectancies in the world. This is thanks to our health system and our model of universal coverage of health care services. The WMA is promoting UHC globally, along with WHO and other international institutions.

